# A Key Role for the Endothelium in NOD1 Mediated Vascular Inflammation: Comparison to TLR4 Responses

**DOI:** 10.1371/journal.pone.0042386

**Published:** 2012-08-01

**Authors:** Timothy Gatheral, Daniel M. Reed, Laura Moreno, Peter J. Gough, Bart J. Votta, Clark A. Sehon, David J. Rickard, John Bertin, Eric Lim, Andrew G. Nicholson, Jane A. Mitchell

**Affiliations:** 1 Cardiothoracic Pharmacology, National Heart and Lung Institute, Imperial College, London, United Kingdom; 2 Department of Pharmacology, School of Medicine, Universidad Complutense de Madrid, Madrid, Spain; 3 Pattern Recognition Receptor Discovery Performance Unit, Immuno-Inflammation Therapeutic Area, GlaxoSmithKline, Collegeville, Philadelphia, Pennsylvania, United States of America; 4 Royal Brompton and Harefield NHS Foundation Trust, London, United Kingdom; 5 National Heart and Lung Institute, Imperial College, London, United Kingdom; 6 Institute of Cardiovascular Medicine and Science (ICMS), London, United Kingdom; Fundação Oswaldo Cruz, Brazil

## Abstract

Understanding the mechanisms by which pathogens induce vascular inflammation and dysfunction may reveal novel therapeutic targets in sepsis and related conditions. The intracellular receptor NOD1 recognises peptidoglycan which features in the cell wall of Gram negative and some Gram positive bacteria. NOD1 engagement generates an inflammatory response via activation of NFκB and MAPK pathways. We have previously shown that stimulation of NOD1 directly activates blood vessels and causes experimental shock *in vivo*. In this study we have used an *ex vivo* vessel-organ culture model to characterise the relative contribution of the endothelium in the response of blood vessels to NOD1 agonists. In addition we present the novel finding that NOD1 directly activates human blood vessels. Using human cultured cells we confirm that endothelial cells respond more avidly to NOD1 agonists than vascular smooth muscle cells. Accordingly we have sought to pharmacologically differentiate NOD1 and TLR4 mediated signalling pathways in human endothelial cells, focussing on TAK1, NFκB and p38 MAPK. In addition we profile novel inhibitors of RIP2 and NOD1 itself, which specifically inhibit NOD1 ligand induced inflammatory signalling in the vasculature. This paper is the first to demonstrate activation of whole human artery by NOD1 stimulation and the relative importance of the endothelium in the sensing of NOD1 ligands by vessels. This data supports the potential utility of NOD1 and RIP2 as therapeutic targets in human disease where vascular inflammation is a clinical feature, such as in sepsis and septic shock.

## Introduction

Sepsis represents a systemic inflammatory response to infection. Severe sepsis is associated with multi-organ dysfunction and may progress to septic shock with hypotension, vascular dysfunction and a high mortality rate of up to 50% [1]. Attempts to ameliorate the host inflammatory response in sepsis, for example with corticosteroids [2] or activated protein C [3] have generated conflicting results in terms of clinical benefit. Indeed, despite initial promising clinical outcome data, activated protein C has now been withdrawn from clinical practise because of poor efficacy [4]. Thus, there remains a need for effective therapies in septic shock to reduce secondary damage to end-organs without compromising pathogen clearance. Accordingly, greater understanding of the interaction between pathogen and host tissue, particularly the vasculature, may reveal relevant therapeutic targets [5], [6].

Invading pathogens are recognised by evolutionally preserved pattern recognition receptors (PRRs), which detect pathogen associated molecular patterns (PAMPs). Identified families of PRRs include the Toll-like receptors (TLRs), nucleotide oligomerisation domain (NOD)-like receptors (NLRs), RIG-1-like receptors (RLRs) and the C-type lectin receptors [7]. Association of PRR and PAMP leads to cell activation by well characterised pathways including induction of nuclear factor kappa-B (NFκB) and inflammatory response genes to promote early immune defences.

Gram negative bacteria are an important cause of sepsis and septic shock. Identified Gram negative PAMPS include lipopolysaccharide (LPS) and peptidoglycan, which are recognised by TLR4 and the NOD receptors respectively [8], [9]. LPS is well known to induce vascular inflammation [10], [11] but less is known about the relative importance of peptidoglycan in driving inflammatory responses [12]. We have previously identified NOD1 as an important receptor mediating vascular inflammation. The addition of the NOD1 agonist FK565 induces vascular dysfunction *in vitro* and mimics septic shock *in vivo* [13]. In another study from our group, the specific vascular effect of NOD1 stimulation was shown to involve signal transduction via NFκB, receptor interacting protein 2 (RIP2) and mitogen activated protein kinases (MAPKs) independently of the inflammasome [14]. In this study we have used a complex whole vessel organ culture system which has allowed us to demonstrate the critical importance of the endothelium in NOD1 sensing, and have translated our original findings in rodent tissues and cells into human tissues. To the best of our knowledge, our study is the first to pharmacologically differentiate NOD1 and TLR4 signalling pathways in vascular cells using highly specific and novel inhibitors of RIP2 and NOD1. This provides the opportunity to dissect the relative roles of NOD1 and TLR4 in more complex models of sepsis and may lead to new therapeutic approaches in septic shock and related microvascular pathologies including acute lung injury.

**Figure 1:**
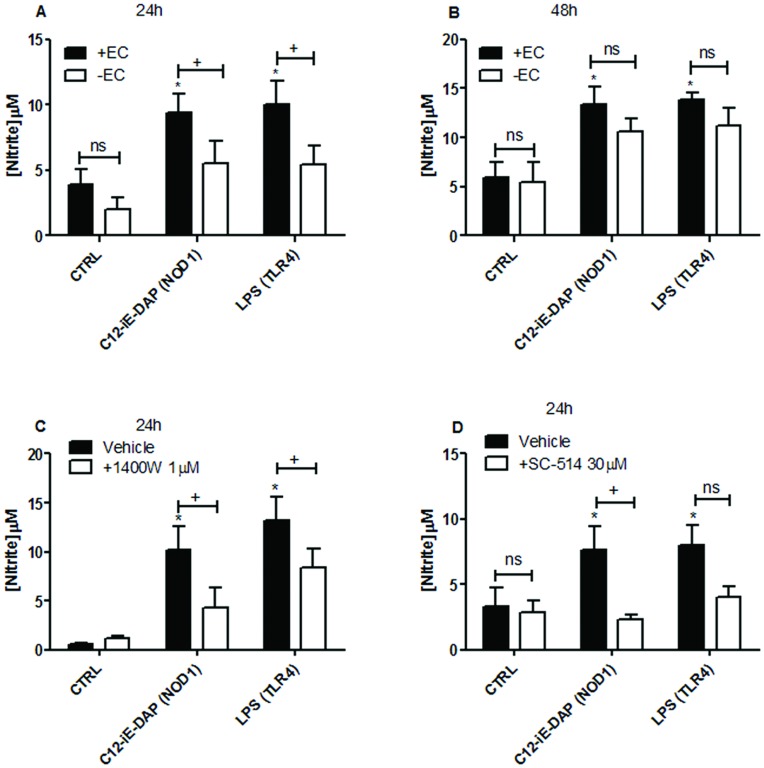
Induction of iNOS activity by NOD1 and TLR4 ligands in whole rat aorta. Aortic rings with endothelium intact (+EC) and denuded (−EC) were treated for 24 hours with media alone (CTRL), C12-iE-DAP 1 µg/ml (NOD1 agonist) or LPS 1 µg/ml (TLR4 agonist) (A) In some experiments, cells were treated for a further 24 hour period with replacement of media and agonists (48 hours; B). In addition, endothelium intact rings were pre-treated with the iNOS inhibitor 1400 W 1 µM (C) or the IκBβ inhibitor SC-514 30 µM (D) for 30 minutes prior to 24 hours treatment with agonists. iNOS activity was assessed by measurement of nitrite (breakdown product of nitric oxide) by the Griess assay. Results are expressed as mean ± SEM for n = 9 (A+B) or n = 4 (C+D) animals. Data was analysed by one-way ANOVA followed by Bonferroni’s Multiple Comparison Test (*P<0.05) or by two-way ANOVA followed by Bonferroni’s post tests (+P<0.05).

**Table 1:** iNOS activity in aortic rings stimulated by the NOD2 agonist MDP.

	Nitrite release (µM)	
	24 hours	48 hours
CTRL	5.9±4.6	10.1±1.9
MDP 10 µg/ml	7.0±1.4	11.7±1.2

iNOS activity was determined by measurement of NO release (via the breakdown product nitrite using the Griess assay). Results are expressed as mean ± SEM for n = 9. Data was analysed by one-way ANOVA followed by Dunnett’s post test.

**Figure 2:**
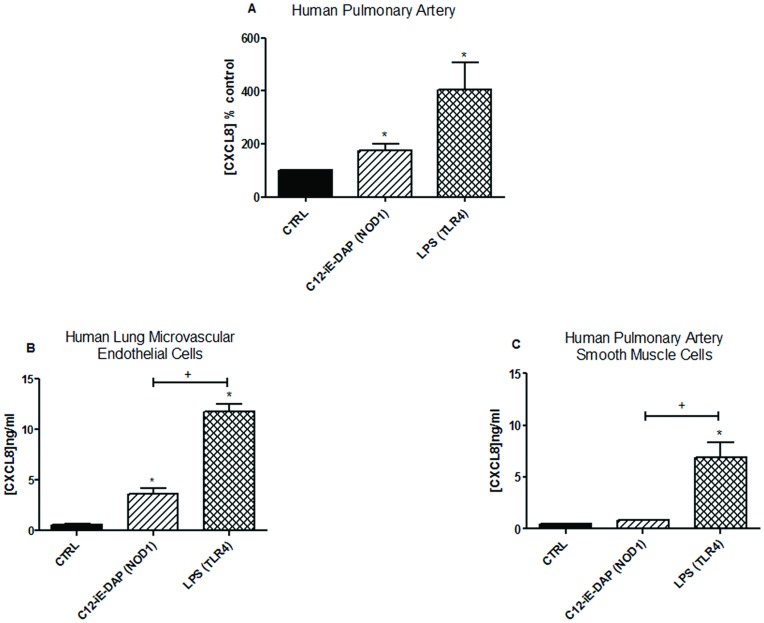
Effect of stimulation with NOD1 and TLR4 ligands on activation of human vascular tissue. (A) Whole human pulmonary artery segments were cultured for 48 hours in the presence of media alone (CTRL), C12-iE-DAP 1 µg/ml (NOD1 agonist) or LPS 1 µg/ml (TLR4 agonist). CXCL8 release was used as a measure of vessel activation. Results are expressed as the percentage (control ± SEM) of CXCL8 release under basal conditions (CTRL) for n = 6 donors. Results were analysed by one-sample t-test (*P<0.05). (B) Human lung microvascular endothelial cells (HMVEC) and (C) Human pulmonary artery smooth muscle cells (HPASMC) were cultured for 24 hours in 96 well plates in the presence of media alone, C12-iE-DAP 1 µg/ml (NOD1) or LPS 1 µg/ml (TLR4). Results are mean ± SEM for n = 12 (HMVEC) or n = 6 (HPASMC). Results were analysed using 1-way ANOVA with Bonferroni’s Multiple Comparison Test. (*P<0.05 *vs*. CTRL), (+P<0.05 *vs*. LPS).

## Methods

### Drugs and Agonists

Two highly specific NOD1 agonists were used in this study, iE-DAP (γ-D-Glu-mDAP) and the acylated derivative C12-iE-DAP (Lauroyl-γ-D-Glu-mDAP) (Invivogen,USA http://www.invivogen.com/). C12-iE-DAP has been reported to display increased cell membrane permeability and potency [15] and was used in organ culture experiments in order to maximise stimulation of NOD1 receptors across the different cell layers. Similarly C12-iE-DAP was used to study NFκB activation at an early time point. Subsequently both agonists were used in cultured human cells. The TLR4 ligand LPS was from Sigma (UK). The inhibitor of NFκB kinase subunit beta (IκBβ) inhibitor SC-514, transforming growth factor β activating kinase (TAK1) inhibitor 5Z-7-oxozeaonol and the pan-caspase inhibitor Z-VAD-fmk were from Tocris Bioscience (UK) and the inducible nitric oxide synthase (iNOS) inhibitor 1400 W from Cayman Chemical (USA). The p38 mitogen activating kinase (p38 MAPK) / receptor interacting protein 2 (RIP2) inhibitor SB203580, the C-jun activating kinase (JNK) inhibitor VI, TI-JIP_153−163_ and the RIP2 / Src kinase inhibitor PP2 were from Merck (Germany) and the p38 MAPK inhibitor BIRB0796 and the extracellular growth factor regulating kinase (ERK) inhibitor PD184352 from Axonmedchem (Netherlands). The RIP2 inhibitor GSK’214 and the NOD1 inhibitor GSK’217 were kindly provided by GlaxoSmithKline pharmaceuticals (USA). Precise structural details for GSK’214 and GSK’217 were not available to us at this time. Primary antibody to the p65 subunit of NFκB, SC-372, was from Santa Cruz (USA). Drugs were dissolved initially in dimethyl sulphoxide (except for iE-DAP, MDP and LPS which were dissolved in PBS) to prepare a 100 or 10 µM stock solution. Further dilutions were made in DMEM. The maximum concentration of DMSO achieved in any experiment, 0.1 %, did not show significant effects on cell viability.

**Table 2:** Effect of the NOD1 agonist iE-DAP on CXCL8 release by HMVEC and HPASM.

	CXCL8 release (% control)	
	HMVEC	HPASM
Control	100±0	100±0
iE-DAP 10 µg/ml	885.6±41.5*	126.8±2.9*

HMVEC and HPASM were cultured for 24 hours in 96 well plates with media alone or iE-DAP 10 µg/ml. Normalised results expressed as mean percentage change from control ± SEM; 12 (HMVEC) or 8 (HPASM). Results were analysed by one-sample t-test (*P<0.05).

**Table 3:** NOD1 and TLR4 ligand induced CXCL8 release in HPASM at 48 hours.

	CXCL8 release ng/ml		CXCL8 release ng/ml		CXCL8 release ng/ml
iE-DAP µg/ml		C12-iE-DAP µg/ml		LPSµg/ml	
CTRL	3.3±0.7	CTRL	3.1±0.1	CTRL	4.0±0.5
0.01	1.9±0.1	0.01	2.1±0.2	0.01	9.7±1.1
0.1	3.7±0.5	0.1	2.3±0.3	0.1	23.5±0.4*
1	2.0±0.2	1	2.8±0.1	1	32.8±2.3*
10	2.2±0.5	10	2.7±0.1	10	23.9±1.0*

HPASM were cultured for 48 hours in 96 well plates with media alone or with iE-DAP (0.01–10 µg/ml), C12-iE-DAP (0.001–1 µg/ml) and LPS (0.001–1 µg/ml). Results are expressed as mean ± SEM for n = 4 from 2 experiments. Results were analysed by one-way ANOVA with Dunnett’s post-test (*P<0.05).

**Table 4:** Effect of NOD1 and TLR4 stimulation on human umbilical vein endothelial cells (HUVEC) and human aortic endothelial cells (HAEC).

	CXCL8 release (% control)	
	HUVEC	HAEC
Control	100	100
IE-DAP 10 µg/ml	473.4±78.7*	216.9±25.9*
LPS 1 µg/ml	1240.0±270.4*	117.7±16.8

HUVEC and HAEC were cultured for 24 hours in 96 well plates with media alone, iE-DAP 10 µg/ml (NOD1 agonist) or LPS 1 µg/ml (TLR4 agonist). CXCL8 release was used as a measure of vessel activation. Normalised results expressed as mean percentage change from control ± SEM for n = 9 (HUVEC) or n = 6 (HAEC). Results were analysed by one-sample t-test (*P<0.05).

**Table 5:** CXCL8 release in HMVEC and HUVEC stimulated by the NOD2 agonist MDP for 24 hours.

	CXCL8 release (% control)	
	HMVEC	HUVEC
CTRL	100	100
MDP 1 µg/ml	100.4±6.9	104.3±11.6
MDP 10 µg/ml	100.7±13.8	107.7±10.8

Normalised results expressed as mean percentage change from control ± SEM for n = 9 (HMVEC) or n = 6 (HUVEC). Results were analysed using a one-sample t-test.

**Table 6:** Inflammatory cytokine and prostacyclin release from HMVEC after stimulation of NOD1 or TLR4.

Analyte (pg/ml)	Treatment		
	CTRL	iE-DAP (NOD1) 10 µg/ml	LPS (TLR4) 1 µg/ml
A			
CXCL8	616.6±79.7	4639.0±365.2*	8712.3±737.6*
GM-CSF	12.5±2.6	93.8±11.7^#^	280.0±50.6^#^
IFNγ	0±0	3.1±0.9*	5.5±0.6*
IL12p70	5.1±2.2	18.4±2.0^#^	76.9±10.6^#^
IL-1β	0.5±0.3	5.4±0.7^#^	7.7±1.0^#^
IL-2	20.8±3.9	141.2±11.8*	258.8±20.5*
IL6	51.1±10.0	111.7±18.2	718.0±89.8*
IP-10	0.5±0.2	1.1±0.3	3.6±0.5*
TNFα	0.6±0.3	2.7±0.6^#^	5.3±1.5^#^
B			
6-keto prostaglandin F1α	318.4±46.3	1322.2±108.2*	2029.3±175.0*

HMVEC were cultured for 24 hours in 96 well plates with media alone (CTRL), iE-DAP 10 µg/ml (NOD1) or LPS 1 µg/ml (TLR4). (A) Inflammatory cytokine release measured using the Human Pro-Inflammatory 9-PlexBase Kit from Meso Scale Discovery (MSD®). Results are expressed as mean ± SEM for n = 15. (B) 6-keto prostaglandin F1α release (breakdown product of Prostacyclin) measured by ELISA. Results are expressed as mean ± SEM for n = 15. Results analysed by one-way ANOVA with Dunnett’s multiple comparison test (*P<0.05) or by Kruskal-Wallis test with Dunn’s multiple comparison test for non-parametric data (^#^P<0.05).

**Figure 3:**
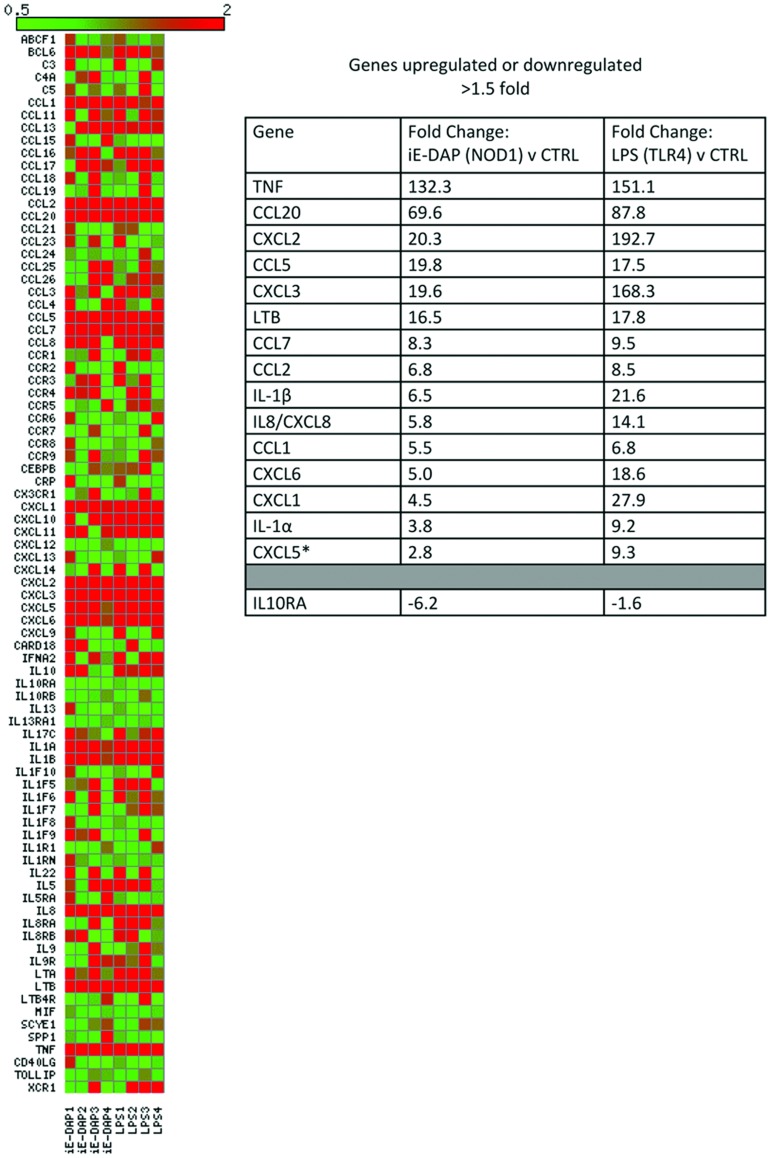
Gene expression pattern in HMVEC following stimulation with NOD1 or TLR4 ligands. Cultured HMVEC were stimulated for 4 hours with iE-DAP 10 µg/ml (NOD1) or LPS 1 µg/ml (TLR4). Pro-inflammatory gene expression was measured using a focussed 84 gene Human Inflammatory Cytokines & Receptors RT2 Profile PCR Array. Results are shown from 2 donors and 4 separate experiments. Overall expression patterns are shown in the heatmap. Genes that were consistently (on each experimental day) increased or decreased by 1.5 fold or above following NOD1 stimulation are shown in the table with the equivalent fold change in response to TLR4 stimulation in the adjacent column. *CXCL5 was induced only 1.4 fold by iE-DAP on the 4th experimental day but this gene was included in the table for comparative purposes.

### Rat Aorta Organ Culture

Rings from rat aortas were prepared as previously described [11]. Briefly male Sprague-Dawley rats (250–450 g) were killed by cervical dislocation following isoflurane anaesthesia. The rats were maintained and killed in accordance with UK Home Office guidelines for the use of experimental animals. Ethical approval and a Home Office project license for the study was not required under United Kingdom Animal (Scientific Procedures) Act of 1986 because mice were killed by cervical dislocation (which is an appropriate method under Schedule 1 of the Act) at a designated establishment. Aortas (thoracic and abdominal) were immediately removed and placed into sterile pots containing phosphate buffered saline (PBS) supplemented with penicillin (1000 U/ml) and streptomycin (1 mg/ml) (PBS; Pen-Strep). Vessels were stored at room temperature for less than 2 h before preparation. For bioassay experiments vessels were dissected clean of connective tissue under sterile conditions then washed 3 times in PBS; Pen-Strep to remove any adherent blood clots. Vessels were then cut into rings of approximately 2–3 mm width. Depending on protocol some of the rings had the endothelium mechanically disrupted by gentle rolling on sterile forceps. Individual rings were then placed into wells of sterile 96-well plates containing 300 µl Dulbecco’s modified Eagle’s medium (DMEM) (GIBCO Life Technologies, UK), supplemented with 10% heat-inactivated foetal calf serum (FCS), L-glutamine (2 mM), streptomycin (100 µg/ml), penicillin (100 U/ml), amphotericin B (2.5 µg/ml) and 1% vol/vol 100×MEM non-essential amino acids (NEAA). All vessels were cultured at 37°C in an atmosphere of 5% CO_2_. Vessels were left to equilibrate for 1 hour prior to replacement with medium containing drugs or vehicle. In selected experiments vessels were pre-treated for 30 minutes with specific cell signalling inhibitors prior to addition of agonists.

Medium was removed at 24 hours for analysis (24 hour time point); fresh medium with treatments was then added for a further 24 hour period (48 hours time point). Replicates from each individual donor were averaged to give one value for subsequent analysis.

### Human Pulmonary Artery Organ Culture

Small segments of pulmonary artery were obtained from lung tissue surplus to diagnostic requirements following thoracic surgery at the Royal Brompton and Harefield NHS Foundation Trust, London, UK (Royal Brompton and Harefield local ethics committee, number 09/H0708172). Full informed written consent was obtained from all participants. The segments were dissected clean of connective tissue and washed 3 times in sterile PBS: Pen-Strep. The segments were carefully dissected into small pieces each approximately 2 mm ×2 mm in surface area. The arterial pieces were then placed in individual wells of a 96 well plate in 300 µl DMEM supplemented as described for the rat aorta organ culture experiments. All vessels were cultured at 37°C in an atmosphere of 5% CO2. Vessel segments were left to equilibrate overnight prior to replacement with medium containing drugs or vehicle. Vessels were treated for a total of 48 hours. Replicates from each individual donor were averaged to give one value for subsequent analysis.

### Cell Culture

Microvascular endothelial cells from the human lung were purchased from 2 companies. Clonetics® ‘human lung microvascular endothelial cells’ (Lonza Walkersville, Inc. Walkersville, MD, USA) and ‘human pulmonary microvascular endothelial cells’ (PromoCell, Heidelberg, Germany). In each case these cells are defined as lung-derived endothelial cells from healthy donors. Two donor populations were used from each company. No differences were observed between both sets of human lung microvascular endothelial cells and, therefore, they will be referred to as human lung microvascular endothelial cells (HMVEC) in this text. Clonetics® human aortic endothelial cells (HAEC) (Lonza) were used from one donor. Human Umbilical Vein Endothelial cells (HUVEC), from at least 2 donors, were kindly donated from the Royal Veterinary College (London, UK). In all cases cells were cultured in 75 cm^2^ sterile culture flasks with appropriate cell culture medium (Table S1). All cells were treated in either 6 or 96 well plates at a seeding density of 1×10^5^ and 8×10^3^ cells/well respectively.

Human pulmonary artery smooth muscle cells (HPASM), from at least 3 donors, were obtained by outgrowth from donor tissue. Human pulmonary artery specimens were previously obtained from healthy segments of lung from patients undergoing lung resection at the Royal Brompton and Harefield NHS Foundation Trust (Research Ethics Committee study number 02–081, sub-amendment 3). Under sterile conditions, the adventitia and endothelium were removed, and the specimens were cut into 3–4 mm^2^ segments and placed in sterile tissue culture flasks. Proliferating smooth muscle cells were subcultured in 75 cm^2^ sterile flasks in DMEM supplemented with 15% FCS, L-glutamine, Pen-Strep and NEAA. Cells were seeded in 96 well plates at a seeding density of 8×10^3^ cells/well. 24 hours prior to treatment, the medium was changed to serum free in order to synchronise growth phase.

Cells were treated for up to 48 hours with the NOD1 agonists iE-DAP (10 µg/ml) and C12-iE-DAP (1 µg/ml), the NOD2 agonist MDP (10 µg/ml) and the TLR4 agonist LPS (1 µg/ml). In certain experiments cells were pre-treated with specific cell signalling inhibitors for 30 minutes prior to the addition of agonists.

### Assessment of Cell Respiration by MTT or AlamarBlue®

Cell respiration, a marker of viability, was assessed after all treatments by either of two methods. Firstly by measuring the reduction of 3-[4,5-dimethylthiazol-2-yl]-2,5-diphenyltetrazolium bromide (MTT) (Sigma, UK) to formazan. Secondly by the alamarBlue® assay (invitrogen, USA) which incorporates a fluorometric/colorimetric oxidation-reduction (REDOX) indicator. No treatment had a significant effect on cell viability.

### Measurement of NO Production

NO production by cultured cells or aortic rings was measured by the accumulation of its oxidation product, nitrite, using the Griess reaction, as we have described previously [16].

**Figure 4:**
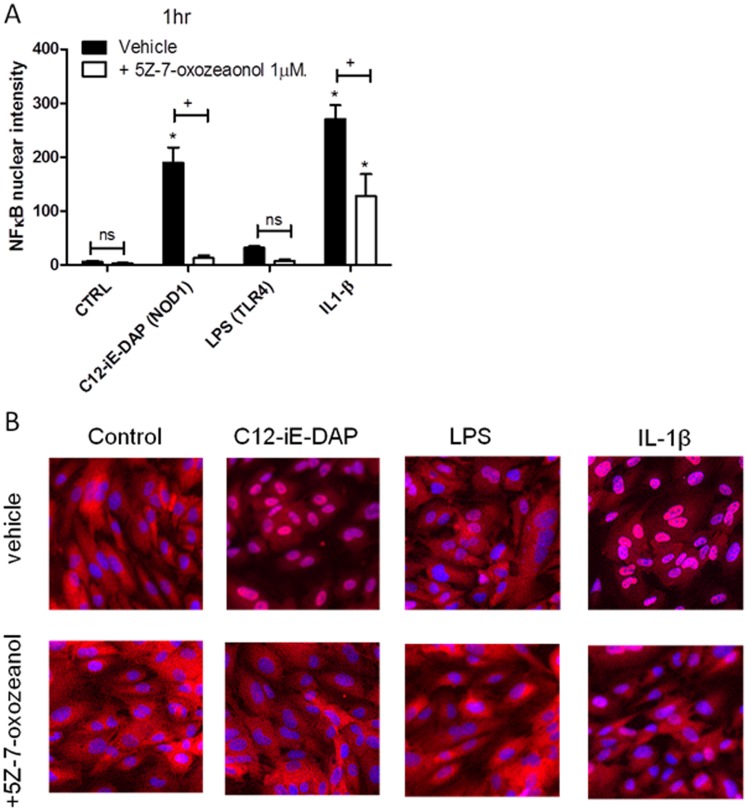
NOD1 or TLR4 ligand and TAK1 mediated NFκB activation in HMVEC. (A) Mean NFκB nuclear intensity/cell (normalised to 1000 cells) following stimulation with C12-iE-DAP 10 µg/ml (NOD1 agonist), LPS 10 µg/ml (TLR4 agonist) or IL-1β 1 ng/ml for 1 hour in the presence or absence of the TAK1 inhibitor 5Z-7-oxozeaonol. Results are mean ± SEM n = 6. Data was analysed by one-way ANOVA followed by Bonferroni’s Multiple Comparison Test (*P<0.05 vs. CTRL) or by two-way ANOVA followed by Bonferroni’s post tests (+P<0.05 vs. vehicle). (B) Representative images of NFκB nuclear translocation under the different experimental conditions.

**Figure 5:**
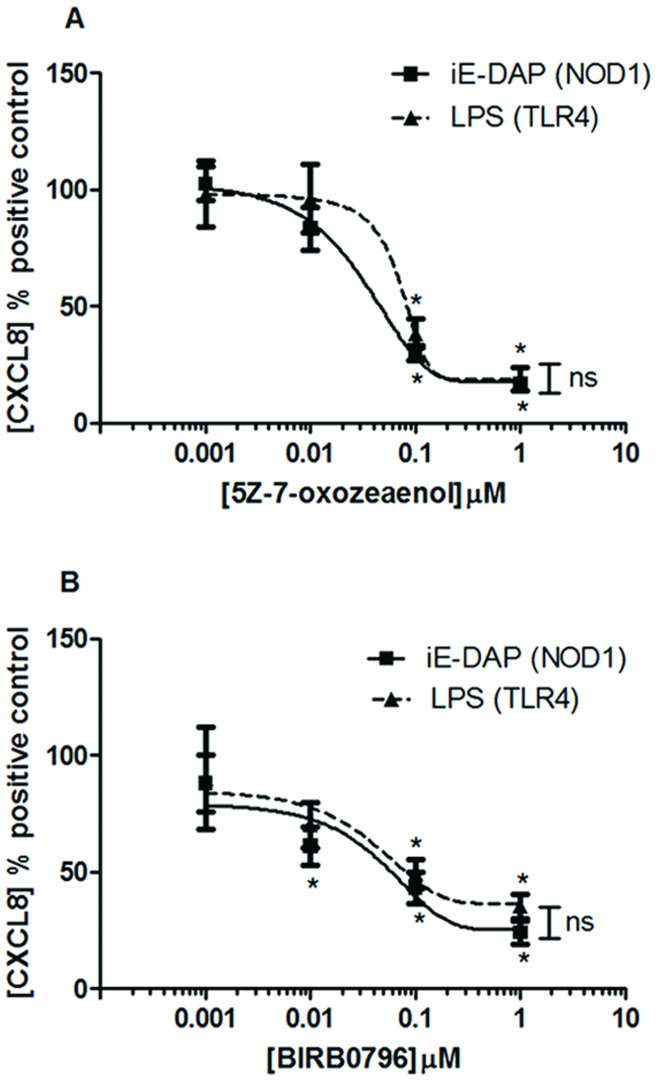
Role of TAK1 and p38 MAPK on NOD1 and TLR4 ligand mediated CXCL8 release by HMVEC. CXCL8 release at 24 hours in HMVEC stimulated by iE-DAP 10 µg/ml (NOD1 agonist) or LPS 1 µg/ml (TLR4 agonist) following 30 minutes pre-treatment with (A) the TAK1 inhibitor 5Z-7-oxozeaenol and (B) the p38 mitogen activated protein kinase (p38 MAPK) inhibitor BIRB0796. Results are expressed as a percentage of response to the agonist alone (mean ± SEM; n = 4). Results were analysed by 1-sample t-test (*P<0.05) and by 2-way ANOVA (#P<0.05 *vs.* LPS).

**Table 7:** Effects of the ERK, JNK and pan-caspase inhibitors PD184352, TI-JIP153-163 and Z-VAD-fmk on NOD1 and TLR4 induced CXCL8 release in HMVEC.

Inhibitor	CXCL8 release (% responseto iE-DAP 10 µg/ml)	CXCL8 release (% responseto LPS 1 µg/ml)
PD184352 (1 µM)	124.7±25.6	103.9±6.1
TI-JIP_153−163_ (30 µM)	109.0±9.4	110.4±7.5
Z-VAD-fmk (10 µM)	93.1±7.3	103.4±13.5

HMVEC were cultured for 24 hours in 96 well plates with media alone (CTRL), iE-DAP 10 µg/ml (NOD1) or LPS 1 µg/ml (TLR4). Signalling inhibitors were added to the cells 30 minutes prior to the addition of agonists. Results are expressed as mean percentage release compared to that induced by the agonists alone (iE-DAP 10 µg/ml or LPS 1 µg/ml) ± SEM for n = 3. Results were analysed by one-sample t-test.

### Analysis of Inflammatory Cytokine Release

Chemokine (C-X-C motif) ligand 8 (CXCL8) release was measured in cell culture supernatants by specific enzyme-linked immunosorbent assay (ELISA) (R&D systems, UK). Release of interferon gamma (IFN-γ), interleukin 2 (IL-2), tumour necrosis factor alpha (TNF-α), interleukin 1-beta (IL-1β), granulocyte-macrophage colony-stimulating factor (GM-CSF), Interferon gamma-induced protein 10 (IP-10), Interleukin 12 p70 subunit (IL-12p70), interleukin 6 (IL-6) and CXCL8 was measured in supernatants from cultured HMVEC using the Meso Scale Discovery® human proinflammatory 9-plex base kit (Meso Scale Discovery, Gaithersburg, MD, USA).

### Measurement of Prostacyclin Release

Prostacyclin release from cells was measured by the breakdown product, 6-keto Prostaglandin F1α, in cell supernatant by specific ELISA (Cayman Chemical Company, Ann Arbor, USA).

### Inflammatory Gene Expression in HMVEC

Total RNA was extracted from cultured HMVEC after 4 hours treatment with NOD1 or TLR4 agonists using the RNeasy Minikit (Qiagen). Gene expression patterns were then analysed using a focussed 84 gene Human Inflammatory Cytokines & Receptors RT2 Profile PCR Array (SABiosciences™, UK) using 243 ng of RNA per array plate. Array data was visualised using the matrix2png program [17].

### Measurement of NFκB Translocation

Following treatment for 1 h cells were washed in PBS and fixed immediately in 4% formaldehyde for 10 mins at room temperature. Plates were then washed three times with PBS at 5 minute intervals. Plates were permeabilized with 0.2% Triton X-100 for 10 minutes and blocked with 4% FCS in PBS for 1 h at room temperature. For NFκB staining, cells were incubated with NFκB-p65 (human) primary antibodies raised in rabbit (Santa Cruz Biotechnology, UK) for 1 h at room temperature followed by secondary staining with AlexaFluor 568 anti-rabbit antibodies raised in goat (Invitrogen, UK) for 45 min at room temperature. Cells were washed three times between incubations with PBS at 5 minute intervals. Cell nuclei were stained with DAPI. Some wells were treated with secondary antibody only in order to determine levels of non-specific background staining. Plates were then stored in PBS at −4°C prior to imaging using a Cellomics VTi HCS Arrayscanner (Thermo Fisher, Pittsburgh, US).

In these experiments the system was used to scan 1000 cells per well across up to 49 fields per well. Images were analysed using a Thermo Fisher compartment analysis bioapplication for cytosol to nucleus translocation [18]. Mean nuclear-cytosolic intensity differences were calculated for each well and normalised to 1000 cells. High nuclear–cytosolic intensity differences corresponded to high nuclear staining and these can be plotted relative to control well values.

### Statistical Analysis

For Cell culture experiments data are mean ± S.E.M for separate incubations and unless otherwise stated protocols were repeated on at least 3 separate experimental days with separately prepared drugs. For organ culture experiments data are mean ± S.E.M for separate animals or individual donors. Non-normalised data was analysed using one or two-way analysis of variance (ANOVA), with Bonferroni post-tests. Normalised data (to control or vehicle) are presented as mean ± SEM and were analysed by one-sample T-test and in some cases by two-way ANOVA with Bonferroni correction. Values of P<0.05 were considered significant.

**Figure 6:**
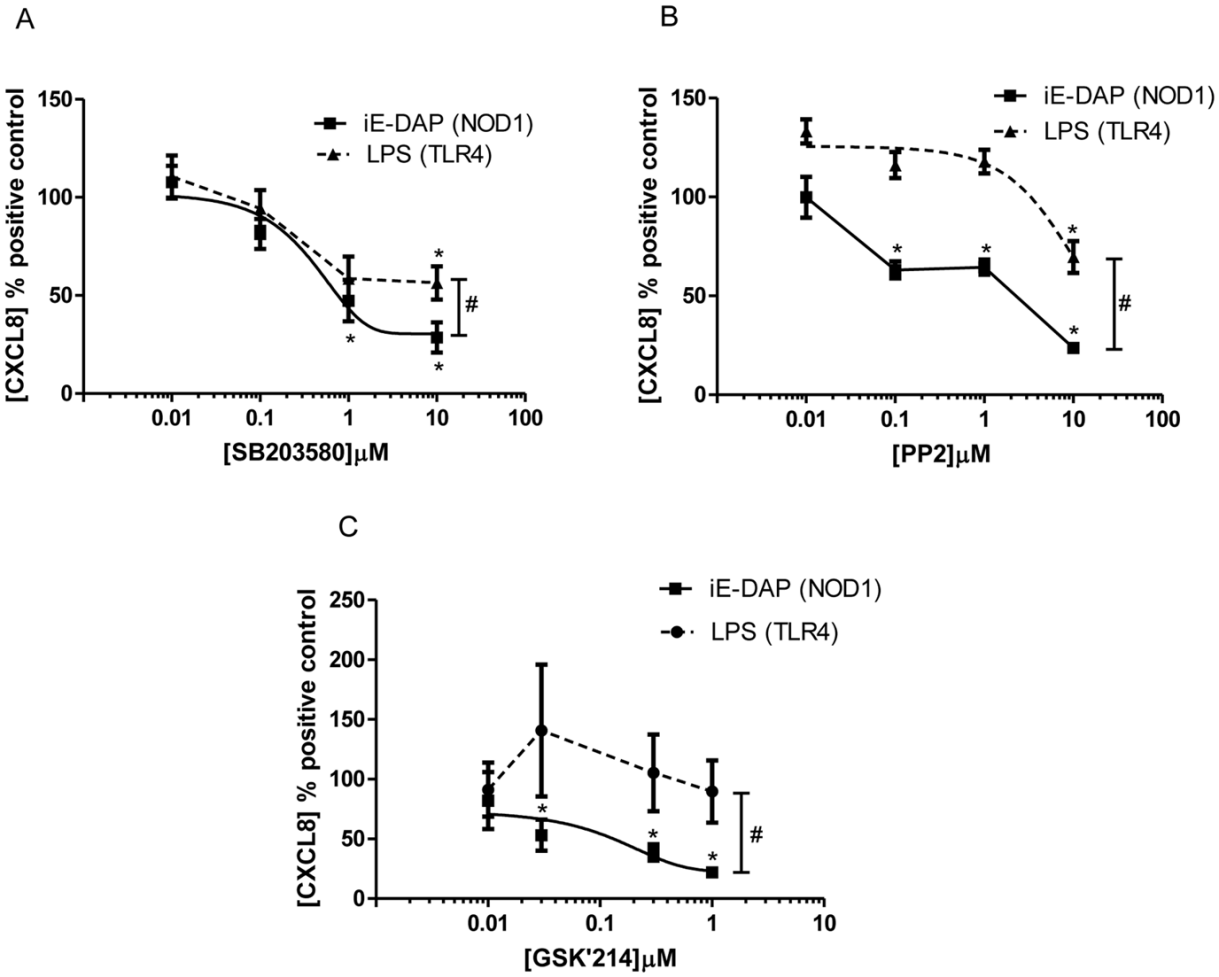
Role of RIP2 on NOD1 and TLR4 ligand mediated CXCL8 release by HMVEC. CXCL8 release at 24 hours in HMVEC stimulated by iE-DAP 10 µg/ml (NOD1 agonist) or LPS 1 µg/ml (TLR4 agonist) following 30 minutes pre-treatment with (A) the receptor interacting protein 2 (RIP2) / p38 MAPK inhibitor SB203580, (B) the Receptor interacting protein (RIP2) / Src kinase inhibitor PP2 and (C) the novel RIP2 inhibitor GSK’214. Results are expressed as a percentage of response to the agonist alone (mean ± SEM; n = 4–6). Results were analysed by 1-sample t-test (*P<0.05) and by 2-way ANOVA (#P<0.05 *vs.* LPS).

**Figure 7:**
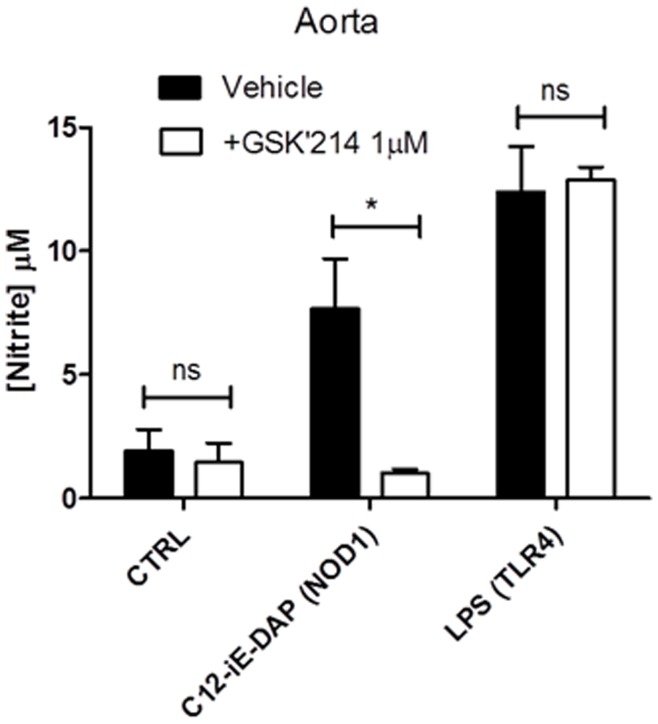
Effect of the novel RIP2 inhibitor GSK’214 on NOD1 and TLR4 ligand mediated iNOS induction by rat vascular tissue. (A) Cultured VSM were cultured for 48 hours in 96 well plates in the presence of media alone or C12-iE-DAP 1 µg/ml (NOD1) following 30 minutes pre-treatment with GSK’214. Results are expressed as a percentage of response to the agonist alone (mean ± SEM; 6). Results were analysed by 1-sample t-test (*P<0.05). (B) Aortic rings with endothelium intact were treated for 24 hours with media alone (CTRL), C12-iE-DAP 1 µg/ml (NOD1 agonist) or LPS 1 µg/ml (TLR4 agonist). Results are expressed as mean ± SEM for n = 3. Results were analysed by 2-way ANOVA with Bonferroni’s post-test (*P<0.05 *vs.* Vehicle).

**Figure 8:**
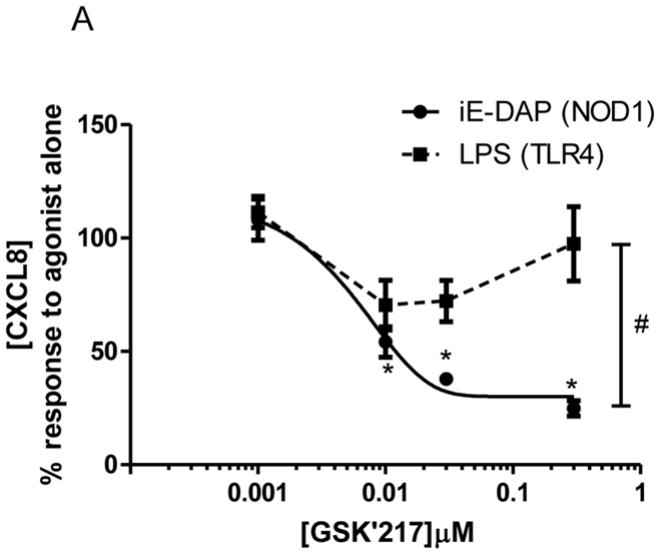
Effect of the novel NOD1 inhibitor GSK’217 on NOD1 and TLR4 ligand mediated CXCL8 release by HMVEC. CXCL8 release at 24 hours in HMVEC stimulated by iE-DAP 10 µg/ml (NOD1 agonist) or LPS 1 µg/ml (TLR4 agonist) following 30 minutes pre-treatment with the novel NOD1 antagonist GSK’217. Results are expressed as a percentage of response to the agonist alone (mean ± SEM; m = 6). Results were analysed by 1-sample t-test (*P<0.05) and by 2-way ANOVA (#P<0.05 *vs.* LPS).

## Results

### Role of the Endothelium in NOD1 or TLR4 Responses in Intact Blood Vessels

As we have previously shown [11], LPS induces iNOS activity, measured by accumulation of nitrite into the culture media, in whole rat aorta in organ culture (Figure 1). In this study we show that the NOD1 agonist C12-iE-DAP similarly induces iNOS activity in whole rat aorta in organ culture (Figure 1). The NOD2 agonist MDP had no effect on iNOS activity (Table 1). In addition, we report here for the first time that, at the 24 h time point, the ability of either LPS or C12-iE-DAP to induce iNOS activity was mediated by the endothelium (Figure 1). The role of the endothelium in responses to LPS or C12-iE-DAP was somewhat lost at the 48 h time point (Figure 1). As expected, and by way of pharmacological validation, the selective iNOS inhibitor 1400 W (1 µM) inhibited nitrite levels induced by either C12-iE-DAP or LPS from whole aorta both at 24 (Figure 1) and 48 hours (not shown). Similarly, iNOS activity induced in whole aorta by either C12-iE-DAP or LPS was found to be critically dependent upon NFκB activation since the IκBβ inhibitor, SC-514 blocked increased nitrite levels induced by either agonist (Figure 1). In order to translate our findings in rat vessels to human tissue we performed additional experiments using intact human vessel (pulmonary artery) segments in the same organ culture system. iNOS is a less prevalent gene in human tissue than in rodent vessels; we therefore used CXCL8 release as a marker of vessel activation. Using this approach we found, as seen in rat aorta, that the NOD1 agonist C12-iE-DAP or the TLR4 agonist LPS induced inflammatory responses, indicated by increased CXCL8 release (Figure 2 + Table S2).

### Effect of NOD1 or TLR4 on Inflammatory Responses in Isolated Human Vascular Cells

The above observations suggest that endothelial cells are important sensors of NOD1 agonists by blood vessels. In order to explore this further we performed additional experiments to compare the ability of human endothelial cells, as well as smooth muscle cells, to sense NOD1 versus TLR4 agonists. At 24 hours, LPS induced robust increases in CXCL8 release from both HMVEC and HPASM (Figure 2). HMVEC released >5 fold increase in CXCL8 in response to the NOD1 agonist C12-iE-DAP (Figure 2). In HPASM however, C12-iE-DAP did not significantly increase CXCL8 release (Figure 2). Consistent with these results the NOD1 agonist iE-DAP (10 µg/ml) induced >8 fold increase in CXCL8 release from HMVEC but failed to increase CXCL8 release by more than 1.5 fold from HPASM (Table 2). Unlike whole aorta, where iNOS induction appears to be shared with the vascular smooth muscle at 48 hours (Figure 1B), HPASM remained unresponsive to a range of NOD1 ligand concentrations at 48 hours (Table 3). In addition to HMVEC we tested the ability of two other human endothelial cells lines to respond to NOD1 agonists. In line with results from HMVEC, HUVEC and HAEC released CXCL8 in response to the NOD1 agonist iE-DAP or the TLR4 agonist LPS (Table 4). In keeping with the organ culture data, the NOD2 agonist MDP failed to induce significant CXCL8 release from HMVEC or HUVEC (Table 5). In addition to CXCL8, iE-DAP or LPS induced increased release of a range of other inflammatory mediators from HMVEC. Thus, stimulation with either iE-DAP (NOD1) or LPS (TLR4) for 24 hours induced the release of CXCL8> IL2> GM-CSF > IL12p70> IL-1β> IFNγ> TNFα (Table 6). iE-DAP also tended to induce IL6 release, although this did not reach statistical significance (Table 6). iE-DAP and LPS similarly induced the release of prostacyclin, the prominent cyclo-oxygenase-derived, vascular lipid mediator, from HMVEC (Table 6). Gene array studies confirmed and validated our observations measuring cytokine protein release. Specifically we found that iE-DAP or LPS induced a remarkably similar gene expression pattern in HMVECs with both PAMPs inducing increased expression of more than 15 inflammatory genes including CXCL8, IL-1β and TNFα (Figure 3). In addition iE-DAP and LPS both downregulated the anti-inflammatory gene interleukin 10 receptor, subunit α (IL10RA). Thus, using our focused array, there was no evidence for NOD1 activation to induce a different set of genes to TLR4.

### Role of RIP2 in NOD1 versus TLR4 Signalling Pathways in HMVECs and Rodent Vascular Tissue

Others have shown that NOD1 receptor signalling occurs via the RIP2, NFκB and TAK1 signalling cascade. TLR4 signals via MyD88 and TRIF dependent pathways linked to NFκB activation. In this study we were able to pharmacologically validate the signalling pathways in human endothelial cells and then use HMVEC as a model system to establish the importance of these signalling pathways to NOD1 and TLR4 responses in the same cell, in experiments performed in parallel. C12-iE-DAP induced NFκB nuclear translocation, an effect that was inhibited by the TAK1 inhibitor 5Z-7-oxozeanenol (Figure 4). LPS induction of NFκB in this particular experimental model is variable so IL-1β was included as a relevant positive control for MyD88 pathway activity downstream of the IL-1 receptor complex. Accordingly IL-1β significantly induced NFκB nuclear translocation, and this effect was also inhibited by 5Z-7-oxozeaonol. In addition, 5Z-7-oxozeaonol inhibited LPS or iE-DAP induced CXCL8 release from HMVEC (Figure 5 + Table S3). In addition, LPS and iE-DAP induced CXCL8 release was inhibited by the p38 MAPK inhibitor BIRB0796 (Figure 5 + Table S3) but not by the ERK, JNK or pan-caspase inhibitors PD184352, TI-JIP_153−163_ or Z-VAD-fmk (Table 7). These observations suggest that in HMVEC, as in other cells, TLR4 or NOD1 pathways induce NFκB via a TAK1 dependent pathway as well as p38 MAPK activity.

Since the vascular inflammatory response driven by NOD1 stimulation in rodents seems to involve RIP2 [14], we next wanted to discriminate TLR4 and NOD1 signalling in human endothelial cells by pharmacological targeting of RIP2. There are a number of compounds available to address this. Using the mixed RIP2/p38 MAPK inhibitor SB203580 we found a moderate selective inhibition of CXCL8 induced by iE-DAP compared to that induced by LPS (Figure 6 and Table S3). We found a clearer selective inhibition of iE-DAP induced CXCL8 release using the commercially available RIP2/Src kinase inhibitor PP2, (Figure 6 and Table S3). Finally, we have used a novel highly selective RIP2 inhibitor, GSK’214, which had no effect on basal CXCL8 release (Table S4) and displayed a striking selectivity towards CXCL8 release by HMVEC induced by the NOD1 agonist iE-DAP, with no effect on LPS induced release (Figure 6 and Table S4). GSK’214 also acted as a highly specific inhibitor of NOD1 ligand induced NO release from whole rat aorta (Figure 7).

### The Novel Antagonist GSK’217 is Active in Human Endothelial Cells and Selectively Inhibits Responses to NOD1, but not TLR4 Ligands

Both NOD1 and NOD2 receptors signal via RIP2 but are activated by different ligands and have diverse roles in inflammatory disease. Research into the relative roles of NOD1 and NOD2 signalling versus other PRRs, including TLR4, has been hampered by the lack of a specific antagonist. We therefore profiled the novel NOD1 inhibitor GSK’217 in HMVEC. GSK’217 acted as a potent and specific inhibitor of iE-DAP mediated CXCL8 release, with no effect on the LPS response (Figure 8 and Table S5).

## Discussion

In this study we have demonstrated that the specific NOD1 ligands iE-DAP and C12-iE-DAP can activate whole vessels without a requirement for co-induction by TLR4. Furthermore our organ culture model suggests that the endothelium is a key sensor for bacterial PAMPs, at least within the first 24 hours of exposure. This technique has been previously used within our group to demonstrate release of key inflammatory mediators including NO and prostanoids from rodent and human vessels [11], [19]. Importantly our study is the first to reveal that the inflammatory effect of NOD1 ligands translates to whole human vessels. The findings in organ culture are supported by in-vitro experiments, in which NOD1 ligands appear to stimulate endothelial cells with greater efficacy than vascular smooth muscle cells. Unlike whole aorta, this endothelial specific effect was maintained at both 24 and 48 hours. We have proceeded to show that stimulation of NOD1 in human endothelial cells induces multiple chemokine and cytokine response genes that are implicated in sepsis including CXCL6, CXCL8, IL1-β and TNFα. It is also important to note, that in endothelial cells, whilst signalling diverges at the level of RIP2, NOD1 and TLR4 ligands induce identical gene/cytokine signatures.

The endothelium has a key role in the pathogenesis of sepsis and its sequelae including acute lung injury (ALI) [20]. Typically, tissue infection/injury leads to inflammatory cytokine production by local immune cells (e.g. macrophages) promoting activation of endothelial cells. This activation is multi-faceted including induction of a pro-thrombotic state, upregulation of adhesion molecules, migration of leucocytes from blood to tissue, increased endothelial layer fluid leak and release of vasoactive mediators including NO, PGI2 and ET-1 [21]. All these effects serve to promote an effective immune response against an invading pathogen but risk damage to host tissue. However previous work by our group and others has demonstrated that stromal cells of the vasculature such as smooth muscle cells can sense pathogens directly without the need for professional immune cells via engagement of TLRs and NOD receptors [13], [16], [22]. With this in mind we have suggested that therapy that targets the vasculature, as opposed to immune cells, may reduce the pathology of sepsis without compromising immunity [5], [6].

Given that the endothelium represents the first point of contact between vessel and blood borne bacteria, TLR4 and NOD1 receptor activity in endothelial cells is likely to be critical in sensing pathogens. Indeed the literature supports an important role for endothelial NOD1 signalling. Responses to the bacteria Chlamydia pneumoniae and the intracellular bacteria Listeria monocytogenes have been shown to be NOD1 dependent in cultured human endothelium [23], [24]. In addition specific NOD1 ligands have been recently demonstrated to activate human coronary artery endothelial cells and induce focal coronary arteritis in a rat model [25]. The data presented in this study corroborates and extends this work by both demonstrating a novel direct effect of NOD1 ligands on intact human vessels and by establishing the relative importance of the endothelium to NOD1 sensing in a whole vessel organ culture model.

The signalling pathways downstream of TLR4 and increasingly of the NOD receptors are well described [6]. Upon LPS binding the TLR4 complex signals via the adaptor protein MyD88 though a Toll/interleukin-1 receptor domain (TIR-TIR) interaction. Subsequent recruitment of IL-1 receptor-associated kinase-1 (IRAK-1), IRAK-4 and TNF receptor associated factor-6 (TRAF-6) leads to physical linkage with TAK1 and the TAK-binding proteins 1,2 and 3 (TAB1,2,3) forming a larger signalling platform [26]. Resulting phosphorylation of TAK1 leads to activation of the IκB kinase (IKK) complex and translocation of NFκB to the nucleus to initiate gene transcription [27]. Alternatively TLR4 signals via TIR domain-containing adaptor molecule-inducing IFN-γ (TRIF) and interferon regulatory factor 3 (IRF-3) to induce NFκB. Signal transduction via NOD1/2 has also been shown to recruit the TAB1/2/3-TAK1 complex [28] leading to NFκB activation. Both TLR4 and NOD1/2 may also induce activation of MAPK including p38 MAPK via NFκB independent pathways [29]. Data presented in this study corroborates these findings in primary human endothelial cells by demonstrating a role for TAK1, NFκB and p38 MAPK in responses to NOD1 or TLR4 ligands. In keeping with our previous work in rat VSM cells [14], NOD1 ligand induced CXCL8 release in HMVEC appeared to be independent of the inflammasome as demonstrated by a lack of inhibition by the pan-caspase inhibitor Z-VAD-fmk.

Our results also support a central role for NFκB in endothelial cell and whole vessel responses to gram-negative PAMPs. C12-iE-DAP induced significant NFκB nuclear translocation at 1 hour in HMVEC (which activates the MyD88 pathway). We have found that the degree of LPS induced NFκB activation in this particular experimental model is variable, even at multiple timepoints, hence we used IL-1β as a positive control for activation of the MyD88 pathway. NFκB is a central pro-inflammatory transcription factor in the aetiology of sepsis [30] therefore our findings add weight to the concept of the NOD1 signalling pathway as a potential target in the development of new therapies.

In our previous *in vivo* study, NOD1 stimulation reproduced the vascular effects of septic shock with hypotension and renal injury but was not shown to induce neutrophil migration into the lung [13]. This suggests that the NOD1 pathway might exert more selective effects on the vasculature than TLR4 during bacterial sepsis and that the capacity to pharmacologically differentiate the signalling pathways of these receptors in models of vascular inflammation may be instructive. RIP2 is well characterised in the literature as a specific downstream signalling protein for NOD1 and NOD2 but not TLR signalling [28], [31], [32], however existing pharmacological inhibitors with activity against RIP2 are relatively non-selective. By using a novel highly specific RIP2 inhibitor, GSK’214, we have been able to selectively dissociate NOD1 and TLR4 pathways in primary human endothelial cells in which both receptors are active, as well as in a whole aorta bioassay. In addition we have demonstrated that a novel, highly specific antagonist of NOD1, GSK’217, can also discriminate between NOD1 and TLR4 activation in primary human endothelial cells. This ‘proof of concept’ data suggests that GSK’214 and GSK’217 may be powerful tools to investigate the relative contribution of Gram-negative PRRs in models of vascular inflammation and raises the possibility of more selective anti-inflammatory drug targeting when attempting to reduce collateral damage from the septic immune response.

It remains unclear how the interplay between individual TLRs and NODs affects the overall immune response but there is evidence to suggest synergistic interaction [33], [34]. Our data however does show that NOD1 stimulation alone induces a significant number of selected pro-inflammatory genes in endothelial cells, with no significant differences to that of TLR4 stimulation. The absence of convincing efficacy from ongoing clinical trials of TLR4 antagonists in gram-negative sepsis [35] emphasises the need to characterise alternative, but complimentary pathways, such as NOD1, to enable the development of new treatments. The fact that our organ culture data suggests that the role of the endothelium is of greater importance in the initial (first 24 hours) response to Gram-negative PAMPs is interesting and possibly suggests delayed activity in the vascular smooth muscle layer. It is beyond the scope of this study to speculate as to how NOD1 and TLR4 mediated inflammation might proceed throughout the blood vessel over longer periods of sepsis. However, key clinical research in the field of sepsis and critical care has established the need for aggressive early intervention in order to minimise later morbidity and mortality [36]. Thus one can hypothesize that modulation of innate immune responses would be most effective in the initial few hours of the septic response, supporting the potential relevance of therapeutic targeting of early NOD1 mediated endothelial inflammation. These questions could be explored by returning to an ‘in-vivo’ model of Gram negative sepsis and the use of the novel selective NOD pathway inhibitors.

## Supporting Information

Table S1 **Culture media and supplements used for various endothelial cell subtypes.**
(DOCX)

Table S2 **NOD1 and TLR4 ligand mediated CXCL8 release from Human Pulmonary Artery at 48 hours (raw data).** Human pulmonary artery was cultured for 48 hours in 96 well plates with media alone (CTRL), C12-iE-DAP 1 µg/ml (NOD1) or LPS 1 µg/ml (TLR4). Results are expressed as mean ± SEM for n = 6 from 6 donors.
(DOCX)

Table S3 **Effect of pharmacological inhibitors on iE-DAP and LPS mediated CXCL8 release from HMVEC at 24 hours (raw data).** HMVEC were cultured for 24 hours in 96 well plates with media alone (CTRL), iE-DAP 10 µg/ml (NOD1) or LPS 1 µg/ml (TLR4). Results are expressed as mean ± SEM for n = 4.
(DOCX)

Table S4 **Effect of novel RIP2 inhibitor GSK’214 on iE-DAP and LPS mediated CXCL8 release from HMVEC at 24 hours (raw data).** HMVEC were cultured for 24 hours in 96 well plates with media alone (CTRL), iE-DAP 10 µg/ml (NOD1) or LPS 1 µg/ml (TLR4). Results are expressed as mean ± SEM for n = 6.
(DOCX)

Table S5 **Effect of the novel NOD1 inhibitor GSK’217 on iE-DAP and LPS mediated CXCL8 release from HMVEC at 24 hours (raw data).** HMVEC were cultured for 24 hours in 96 well plates with media alone (CTRL), iE-DAP 10 µg/ml (NOD1) or LPS 1 µg/ml (TLR4). Results are expressed as mean ± SEM for n = 6.
(DOCX)
