# Supplementary material for: A Key Role for the Endothelium in NOD1 Mediated Vascular Inflammation: Comparison to TLR4 Responses
Source: PLoS One. 2012 Aug 1;7(8):e42386. doi: 10.1371/journal.pone.0042386 (PMC3411636; doi:10.1371/journal.pone.0042386)
Supplement: Table S1 — Culture media and supplements used for various endothelial cell subtypes. (DOCX) [file pone.0042386.s001.docx]

| Endothelial cell subtype | Media | Supplements |
| --- | --- | --- |
| HMVEC (Lonza) | EBM-2 basal medium (Lonza) | EGM-2 MV SingleQuots (Lonza) containing 25mls FBS (5%), 0.5ml hEGF, 2.0ml hFGF-B, 0.5ml VEGF, 0.5ml ascorbic acid, 0.2ml hydrocortisone, 0.5ml Long R3-IGF-1, 0.5ml heparin and 0.5ml gentamycin/amphotericin. |
| HMVEC (Promocell) | Endothelial Cell Growth Medium-MV2 (Promocell) | 5% FCS, EGF 5ng/ml, FGF 10ng/ml, Long R3 IGF-1 20ng/ml, VGEF 0.5ng/ml, Ascorbic acid 1µg/ml and hydrocortisone 0.2 µg/ml. |
| HUVEC | Medium 199 (Sigma) | 20% FCS, EGF, Pen-Strep and heparin 45µg/ml. |
| HAEC | EBM-2 basal medium (Lonza) | EGM-2 SingleQuots (Lonza). As for EGM-1 MV but only 10mls FBS (2%). |
