# Supplementary material for: A Key Role for the Endothelium in NOD1 Mediated Vascular Inflammation: Comparison to TLR4 Responses
Source: PLoS One. 2012 Aug 1;7(8):e42386. doi: 10.1371/journal.pone.0042386 (PMC3411636; doi:10.1371/journal.pone.0042386)
Supplement: Table S2 — NOD1 and TLR4 ligand mediated CXCL8 release from Human Pulmonary Artery at 48 hours (raw data). Human pulmonary artery was cultured for 48 hours in 96 well plates with media alone (CTRL), C12-iE-DAP 1 µg/ml (NOD1) or LPS 1 µg/ml (TLR4). Results are expressed as mean ± SEM for n = 6 from 6 donors. (DOCX) [file pone.0042386.s002.docx]

| PAMP | CXCL8 release ng/ml |
| --- | --- |
| CTRL | 106.3 ± 20.5 |
| C12-iE-DAP 1µg/ml (NOD1) | 160.0 ± 25.7 |
| LPS 1µg/ml (TLR4) | 278.7 ± 35.0 |
