# Supplementary material for: A Key Role for the Endothelium in NOD1 Mediated Vascular Inflammation: Comparison to TLR4 Responses
Source: PLoS One. 2012 Aug 1;7(8):e42386. doi: 10.1371/journal.pone.0042386 (PMC3411636; doi:10.1371/journal.pone.0042386)
Supplement: Table S3 — Effect of pharmacological inhibitors on iE-DAP and LPS mediated CXCL8 release from HMVEC at 24 hours (raw data). HMVEC were cultured for 24 hours in 96 well plates with media alone (CTRL), iE-DAP 10 µg/ml (NOD1) or LPS 1 µg/ml (TLR4). Results are expressed as mean ± SEM for n = 4. (DOCX) [file pone.0042386.s003.docx]

| Inhibitor µM | CXCL8 release ng/ml | | |
| --- | --- | --- | --- |
|  | CTRL | iE-DAP 10µg/ml | LPS 1µg/ml |
| 5Z-7-oxozeaonol (TAK1) |  | | |
| Vehicle | 3.4 ± 0.7 | 11.4 ± 1.9 | 17.0 ± 4.0 |
| 0.001 | 3.7 ± 0.7 | 3.7 ± 1.4 | 15.3 ± 1.6 |
| 0.01 | 2.7 ± 0.3 | 2.7 ± 1.1 | 14.8 ± 1.2 |
| 0.1 | 1.8 ± 0.3 | 1.8 ± 0.5 | 6.0 ± 0.7 |
| 1 | 1.3 ± 0.2 | 1.3 ± 0.3 | 2.7 ± 0.3 |
| BIRB0796  (p38 MAPK) |  |  |  |
| Vehicle | 2.4 ± 0.5 | 11.1 ± 2.1 | 19.8 ± 2.8 |
| 0.001 | 2.2 ± 0.4 | 9.5 ± 1.8 | 16.3 ± 4.3 |
| 0.01 | 1.4 ± 0.2 | 6.6 ± 1.3 | 13.0 ± 1.4 |
| 0.1 | 0.9 ± 0.2 | 4.6 ± 0.9 | 9.3 ± 1.3 |
| 1 | 0.9 ± 0.2 | 2.7 ± 0.7 | 6.8 ± 1.3 |
| SB203580  (p38 MAPK/RIP2) |  |  |  |
| Vehicle | 2.4 ± 0.6 | 8.9 ± 1.3 | 16.6 ± 1.7 |
| 0.01 | 2.4 ± 0.3 | 9.4 ± 1.3 | 17.8 ± 1.2 |
| 0.1 | 1.4 ± 0.3 | 7.1 ± 1.1 | 15.4 ± 1.7 |
| 1 | 1.0 ± 0.2 | 4.0 ± 0.9 | 9.6 ± 1.9 |
| 10 | 1.0 ± 0.2 | 2.5 ± 0.7 | 9.0 ± 1.1 |
| PP2  (Src kinase/RIP2) |  |  |  |
| Vehicle | 0.9 ± 0.3 | 13.7 ± 1.0 | 22.5 ± 1.6 |
| 0.01 | 1.4 ± 0.1 | 13.6 ± 1.4 | 30.0 ± 2.9 |
| 0.1 | 1.4 ± 0.1 | 8.6 ± 0.3 | 25.8 ± 0.9 |
| 1 | 2.2 ± 0.1 | 8.8 ± 0.6 | 26.2 ± 0.7 |
| 10 | 1.0 ± 0.1 | 3.2 ± 0.1 | 15.5 ± 1.7 |
