# Supplementary material for: A Key Role for the Endothelium in NOD1 Mediated Vascular Inflammation: Comparison to TLR4 Responses
Source: PLoS One. 2012 Aug 1;7(8):e42386. doi: 10.1371/journal.pone.0042386 (PMC3411636; doi:10.1371/journal.pone.0042386)
Supplement: Table S5 — Effect of the novel NOD1 inhibitor GSK’217 on iE-DAP and LPS mediated CXCL8 release from HMVEC at 24 hours (raw data). HMVEC were cultured for 24 hours in 96 well plates with media alone (CTRL), iE-DAP 10 µg/ml (NOD1) or LPS 1 µg/ml (TLR4). Results are expressed as mean ± SEM for n = 6. (DOCX) [file pone.0042386.s005.docx]

| Inhibitor µM | CXCL8 release ng/ml | | |
| --- | --- | --- | --- |
|  | CTRL | iE-DAP 10µg/ml | LPS 1µg/ml |
| GSK’217  (NOD1) |  |  |  |
| Vehicle | 5.0 ± 0.7 | 13.3 ± 0.8 | 17.5 ± 1.9 |
| 0.001 | 4.5 ± 0.8 | 14.4 ± 1.4 | 19.1 ± 1.3 |
| 0.01 | 4.5 ± 1.0 | 7.0 ± 0.6 | 11.4 ± 1.0 |
| 0.03 | 5.6 ± 1.0 | 4.9 ± 0.1 | 11.9 ± 0.7 |
| 0.3 | 5.1 ± 0.9 | 3.1 ± 0.3 | 15.2 ± 2.0 |
